# PPInS: a repository of protein-protein interaction sitesbase

**DOI:** 10.1038/s41598-018-30999-1

**Published:** 2018-08-20

**Authors:** Vicky Kumar, Suchismita Mahato, Anjana Munshi, Mahesh Kulharia

**Affiliations:** 1 Department of Computational Sciences, School of Basic and Applied Sciences, Central University of Punjab, Bathinda, Punjab 151 001 India; 2 Department of Human Genetics and Molecular Medicine, School of Health Sciences, Central University of Punjab, Bathinda, Punjab 151 001 India

## Abstract

**P**rotein-**P**rotein **In**teraction **S**itesbase (PPInS), a high-performance database of protein-protein interacting interfaces, is presented. The atomic level information of the molecular interaction happening amongst various protein chains in protein-protein complexes (as reported in the Protein Data Bank [PDB]) together with their evolutionary information in Structural Classification of Proteins (SCOPe release 2.06), is made available in PPInS. Total 32468 PDB files representing X-ray crystallized multimeric protein-protein complexes with structural resolution better than 2.5 Å had been shortlisted to demarcate the protein-protein interaction interfaces (PPIIs). A total of 111857 PPIIs with ~32.24 million atomic contact pairs (ACPs) were generated and made available on a web server for on-site analysis and downloading purpose. All these PPIIs and protein-protein interacting patches (PPIPs) involved in them, were also analyzed in terms of a number of residues contributing in patch formation, their hydrophobic nature, amount of surface area they contributed in binding, and their homo and heterodimeric nature, to describe the diversity of information covered in PPInS. It was observed that 42.37% of total PPIPs were made up of 6–20 interacting residues, 53.08% PPIPs had interface area ≤1000 Å^2^ in PPII formation, 82.64% PPIPs were reported with hydrophobicity score of ≤10, and 73.26% PPIPs were homologous to each other with the sequence similarity score ranging from 75–100%. A subset “Non-Redundant Database (NRDB)” of the PPInS containing 2265 PPIIs, with over 1.8 million ACPs corresponding to the 1931 protein-protein complexes (PDBs), was also designed by removing structural redundancies at the level of SCOP superfamily (SCOP release 1.75). The web interface of the PPInS (http://www.cup.edu.in:99/ppins/home.php) offers an easy-to-navigate, intuitive and user-friendly environment, and can be accessed by providing PDB ID, SCOP superfamily ID, and protein sequence.

## Introduction

Proteins are the biomolecular substance which are responsible for a large number of cellular processes like catalysis of biochemical reactions, transportation of molecules, synthesis and repair of DNA molecules, etc. in living organisms. Considering their inability to perform in isolation, proteins interact with other molecules like protein, DNA, lipid, etc., and forms the supramolecular entities and carries out most of these biological functioning in living beings. Among all type of complexes that proteins form, protein-protein complexes have attracted the attention of a wide range of scientific community to decipher their underlying principles, role in the various biological phenomenon, and applicability in therapeutic strategies. Consequently, a substantial amount of experimental data, attributed to the technological advancement, pertaining to the protein-protein interactions (PPIs) has been made available. Several attempts have been made by people to organize this PPI data. As a result, databases like 3D Interlogs^1^ (stores the evolutionary lineages of protein); comPPI^2^ (details specific subcellular locations of proteins); PINT^3^ (collection of thermodynamic parameters such as free energy change, enthalpy change, heat capacity change etc., upon binding) etc., have come up. Another approach has been to combine the experimentally gathered interaction data (generated by the application of high-throughput techniques) to identify the protein-protein complexes and databases like GRID^4^; DIP^5^; IntAct^6^; BIND^7^; MINT^8^; HPRD^9^; and STRING^10^ store such information. These databases have also been combined to evolve a more participative approach, such as HitPredict^11^, with a multitude of protocols to quantitatively score the PPI. Some groups have also looked at the residue-based interactions, by employing a “distance dependent atom contact” definition, to look at the “set of interacting residues”. Databases like HotRegion^12^, JAIL^13^, SNAPPI-DB^14^, etc. have considered “interacting interface residue set” as a more apt parameter for characterization of PPIs.

Development of such databases has huge applications in designing computational approaches for PPI sites prediction. Information derived by analysing existing PPIs in terms of protein sequences, structural information, evolutionary conservation, binding-energy, etc., provides scientific facts that need be considered in formulating novel computational strategies. For example, patch analysis based approaches of Jones and Thornton^15^, Ofran and Rost^16^, and Murakami and Jones^17^ were designed by analysis the dataset of known proteins-protein complexes from structural perspectives. The approach given by Aytuna *et al*.^18^ was emphasized on the structure and sequence conservation based features of PPI sites retrieved from known protein complexes. Sequence profile of proteins was assessed by Zhou and Shan^19^ and Shen *et al*.^20^ for their ability to function as protein-protein interaction descriptor. “Weighted sparse representation” based classification and the concept of global encoding was studied in a protein sequence-oriented approach of Huang *et al*.^21^. Evolutionary information of known PPI sites was examined in conjunction with machine learning approaches like neural network^22^ and random forest^23^. Newer concepts in machine learning approaches *viz*. “relevance vector machine”^24^, “discriminative vector machine”^25^, and “rotation forest based classifier”^26^ were also introduced to get new perspective from the protein evolutionary information. In addition to these, hot spot residues-based^27^, knowledge-based^28^, and ensemble approaches^29,30^ were also devised by examining the information derived from the known protein-protein interactions.

In this article, we are presenting the Protein-Protein Interaction Sitesbase (PPInS), a database with high-performance and one of the largest collection, to the best of our knowledge, of protein-protein interaction interfaces (PPIIs). Each PPII is linked to its cognate SCOP superfamily pair. PPInS is an advancement over all existing databases as it not only provides a clear demarcation of PPIIs but also gives information like number and type of residues (regarding interacting, non-interacting and missing residues), SCOP superfamily of interacting patches, and the three-dimensional structural representation of complex under the study. It also covers a vast diversity of protein-protein interaction patches (PPIPs) in terms of a number of residues involved in PPIP formation, their hydrophobicity level, homo and heterodimeric nature of interacting PPIPs, and the amount of surface of the PPIPs devoted to PPII formation. A smaller subset of PPInS is also designed by removing all structural redundancies at the level of SCOP superfamily (SCOP release 1.75).

## Designing of PPInS

### Construction of the database of PPIIs: PPInS

**Figure 1:**
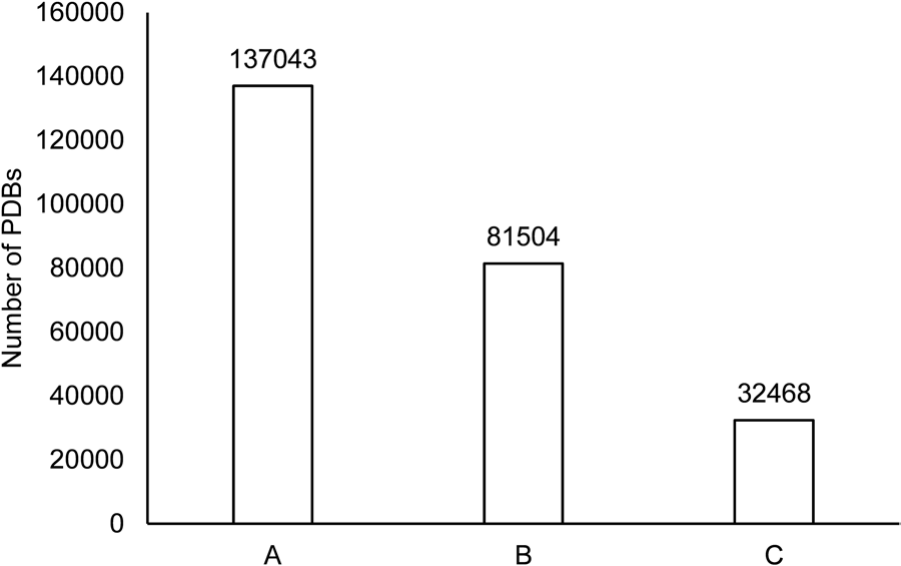
Protein-protein complexes covered in the creation of PPInS. A: Total PDBs, B: PDBs reported in SCOPe 2.06, C: PDBs covered in PPInS.

From over 130 K structural files (PDBs^31^), around 81 K PDBs for which the information of structural classification was reported in SCOPe^32^ (version 2.06; released on 27^th^ Oct 2016), were processed. Only X-ray crystallized multimeric protein-protein complexes with structural resolution better than 2.5 Å were retained for this work. Further, to ensure that only experimentally validated protein structures were used, PDBs representing homology models, other than X-ray crystallography-based structures, nucleic acids and multi-model structures were discarded. At the end, we were left with 32468 PDBs (Fig. 1). The average resolution of retained protein-protein complexes was ~2 Å. For these 32468 PDBs, the interacting atoms between each unique pair of protein chains of each PDB were demarcated.

**Figure 2:**
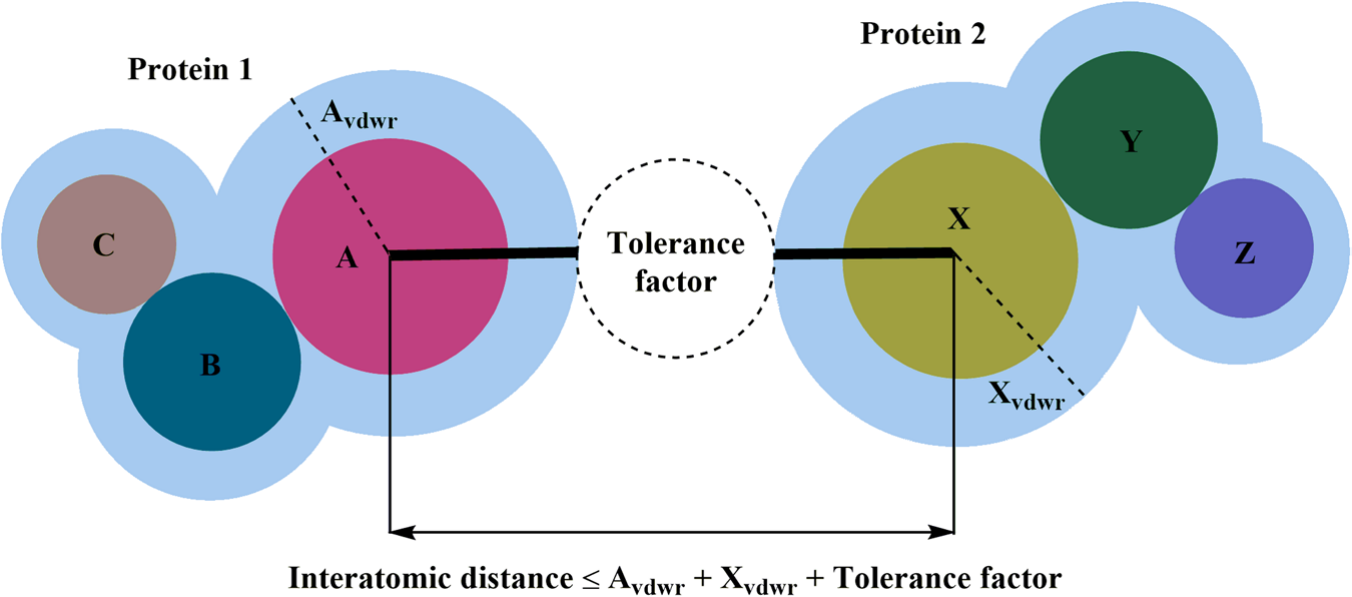
Definition of an atomic contact pair (ACP).

Two atoms belonging to two different protein chains of a PDB were considered to be in contact and demarcated as an atomic contact pair (ACP) if the intervening distance between them was less than the sum of their van der Waals radii plus 1 Å as tolerance factor (Fig. 2). Tolerance factor was incorporated to compensate for the structural aberrations by virtue of resolution and/or thermal fluctuations. A similar definition of interatomic atomic contact was earlier used by Conte *et al*.^33^, Sol and O’Meara^34^ (with a tolerance limit of 0.5 Å), and Kulharia *et al*.^35^. Another type of distance criteria for demarcating the PPI has been to use a sphere of fixed radius (generally 5 or 6 Å)^36,37^ centered on the interacting atoms. Here we have used the atom specific distance criteria wherein the threshold value to determine the presence or absence of interaction is calculated by taking into account the type of atoms. The collection of ACPs between a pair of interacting protein chains was referred as “**P**rotein-**P**rotein **I**nteraction **I**nterface” (PPII). For the notational purpose, atoms from each protein chain involved in PPII formation were collectively termed as a protein-protein interaction patch (PPIP). With these definitions, a total of 111857 PPIIs, with around 32 million ACPs in them, were generated from the 32468 PDBs and given the name of Protein-Protein Interaction Sitesbase (PPInS).

### Construction of a non-redundant subset of PPInS: NRDB

**Figure 3:**
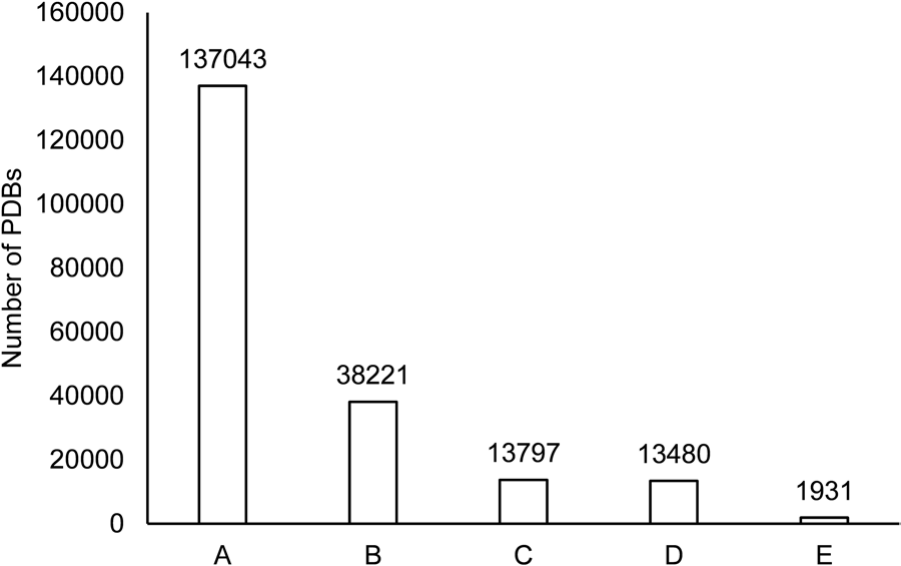
PDBs covered in the creation of NRDB. A: Total protein complexes in PDB, B: PDBs reported in SCOP 1.75, C: PDBs selected for PPII generation, D: PDBs for which PPIIs were generated, E: PDBs covered in NRDB.

NRDB is a subset of PPInS. It was created by removing all structural redundancies at the level of SCOP superfamily. The manually curated SCOP^38^ version 1.75 was used which had structural classification data for 38221 PDBs. Using the same filtering approach as mentioned in designing of PPInS, 13797 PDBs were shortlisted for the construction of NRDB (Fig. 3). For these 13797 PDBs, ACPs between each unique pair of protein chains of all the shortlisted PDBs were demarcated. In this version of PPIIs collections, PPIIs with more than 20 ACPs were considered only. There were 317 such PDBs that could not fulfil this criterion. Therefore, a total of 43509 PPIIs were identified with respect to 13480 PDBs. Further, to make this collection of 43509 PPIIs a non-redundant, a cognate “SCOP superfamily pair” (representing the SCOP superfamily of interacting chains in PPII) was assigned to each PPII. The redundancy was reduced at the structural level by selecting only one PPII (one with the maximum number of ACPs) for each SCOP superfamily pair as a part of NRDB. This was done to maximize the information content of NRDB. The NRDB thus formed contained a total of 2265 unique SSP-based PPIIs, demarcated from 1931 PDB files and representing 43509 PPIIs and SCOP superfamily pairs.

## PPInS Implementation

**Figure 4:**
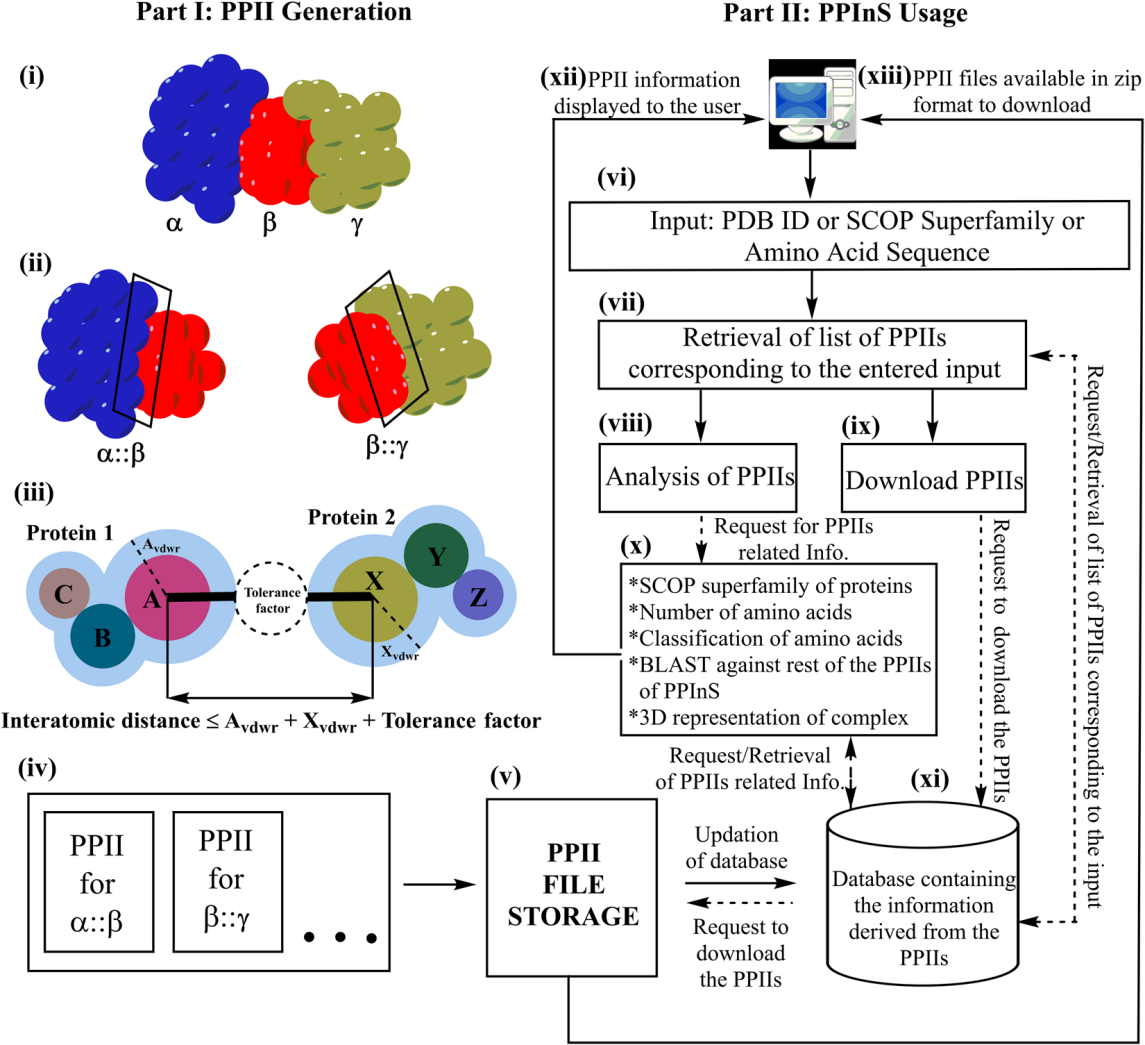
Overview of PPInS.

Web server for PPInS (http://www.cup.edu.in:99/ppins/home.php) was designed using php scripts and Apache web server. The complete overview of PPInS implementation is given in Fig. 4. Part I of the figure describe the process of PPII generation for a hypothetical ternary complex containing three protein chains (α, β, γ). The initial step requires separation of the ternary complex into its constituent binary components (say α::β and β::γ) followed by the identification of interacting amino acids from each of its constituent binary components to fetch the atomic details of the rectangle portion of Fig. 4(ii). The concept of ACP, given in Fig. 4(iii), was used identify the interacting atoms. The collection of obtained ACPs for each pair of protein chains is stored in the form of files (PPII files) containing the atomic details of proteins chains involved in interaction and the interatomic distance between the interacting atoms pairs Fig. 4(iv). All of the PPIIs were made a part of “PPII File Storage” to make them available for downloading purpose. The information derived from these PPIIs is used to create a local database of PPIIs containing information like SCOP superfamily of the interacting protein chains, number of amino acids in each interacting chains and role of amino acids involved.

Part II of the figure describes the stepwise usage of PPInS. On receiving a suitable input from the user in the form of a four-letter alphanumeric PDB ID or a five-digit SCOP superfamily or protein sequence in fasta format. For a PDB ID or SCOP ID based input, PPInS retrieve the list of PPIIs available for the entered input from the local database. Subsequent to this, PPInS gives the option of downloading the retrieved PPIIs and their on-site analysis. The request from the user to download the PPIIs is served by providing the requested PPII files from the “PPII File Storage” (flat files stored in a computer system) in a downloadable format to the user. For on-site analysis of PPIIs, information like cognate SCOP superfamily of the interacting protein chains in the PPIIs, number of amino acids in interacting chains and their role in PPII (in terms of interacting, non-interacting and missing or unknown), are fetched from the local database and displayed to the user. A feature to compare the query protein sequence of a given PPII against the database of protein sequences of PPInS, using BLAST^39^ algorithm, is also provided which allows the user to analyze the PPIIs in which similar kind of protein chain is playing the role of interacting partner. The three-dimensional view of the protein-protein complex under study is also provided using JSmol online viewer.

PPInS also provide for an additional utility, to identify close homologs for a given protein sequence that participates in formations of PPIIs. To improve the speed for the homolog detection, all protein chains from the known three-dimensional complexes included in PPInS are clustered on the basis of their sequence similarity using CD-HIT^40^ algorithm. We have kept the threshold for sequence similarity as ≥90% to ensure only close homologs are clustered in one group. For each cluster, a cluster head (or cluster representative) is designated amongst the cluster members by CD-HIT in such a manner that the cognate cluster members have sequence similarity of ≥90% to the cluster head. In this way, a total of 13190 clusters were produced. The protein sequence entered by user is aligned with cluster heads of these 13190 clusters and cluster heads with ≥90% sequence similarity are presented to the user. Such cluster heads, along with their cluster members, are seen participating as PPIPs in PPII formation, therefore, these clusters heads are provided to user in the forms of PPII for downloading purpose. Two case studies describing the usage of PPInS is also provided here.

### Case Study 1: When a PDB ID is searched for PPIIs

Let us consider the PDB ID “150 L” (structural model for “Conservation of Solvent-Binding Sites in 10 Crystal Forms of T4 Lysozyme”) as an input. This file has a pentanary complex which can be deconvoluted as five binary protein-protein complexes *viz*. A_B_150 L.int, A_C_150 L.int, B_C_150L.int, B_D_150L.int, and C_D_150L.int, representing the PPIIs between chains “A&B”, “A&C”, “B&C”, “B&D” and “C&D”, respectively. The user has an option of downloading these PPIIs files selectively or collectively in zip format by clicking on the “Download” button. The user can also access more information on PPII by clicking on “More Info” button given next to each PPII, which includes:

#### SCOP Superfamily

The SCOP superfamily identity of both the protein chains involved in PPII is displayed to the user. For example, in the current instance of PDB “150L”, each of its protein chains participating in its five PPIIs belong to SCOP superfamily ID 53955. It is also possible for a protein chain to have multiple SCOP superfamily identities i.e. when one domain of protein belongs to one SCOP superfamily while a different SCOP superfamily is reported for another domain.

#### Amino acid sequences

The amino acid sequences of the chains involved in a PPII is also displayed to the user. The role of each amino acid in the formation of PPII is depicted by a coloring scheme. The amino acids are marked red, blue and pink representing the “interacting”, “non-interacting” and “missing or unknown amino acids”, respectively.

#### Sequence similarity of a protein chain

The user has an option to identify proteins similar in sequence (hence also in function) to a given protein. This feature is a method for the user to look for the homologous protein-protein complex interface. Each protein chain is compared for sequence similarity, using BLAST, against the comprehensive database of interacting protein chains from which the PPInS has been derived. The results are displayed in a format of <chain one>_<chain two>_<PDB code>. For example “A_C_150L” specifies that “A” chain of the PPII between protein chains A and C of 150L is similar to the queried protein chain. The extent of sequence similarity for these two protein chains (i.e., queried and target protein chain from PPInS), is also mentioned as a part of the BLAST result. The PPIIs A_C_150L and C_A_150L both are same, and only the former exists in the PPInS.

#### 3D-view of protein complex

The three-dimensional view of protein structure using the JSmol online viewer.

### **Case** Study 2: When a SCOP Superfamily is searched for PPIIs

On inputting SCOP superfamily “63707” to the PPInS, a list of 10 PPIIs will be displayed with an option to download them. Each of these 10 PPIIs has at least one interacting protein chain which belongs to SCOP superfamily “63707”. All the functions that are discussed for the retrieved PPIIs in case study 1 are also applicable here too.

## Diversity of information contained in PPInS

### Number of residues involved in PPIPs

**Figure 5:**
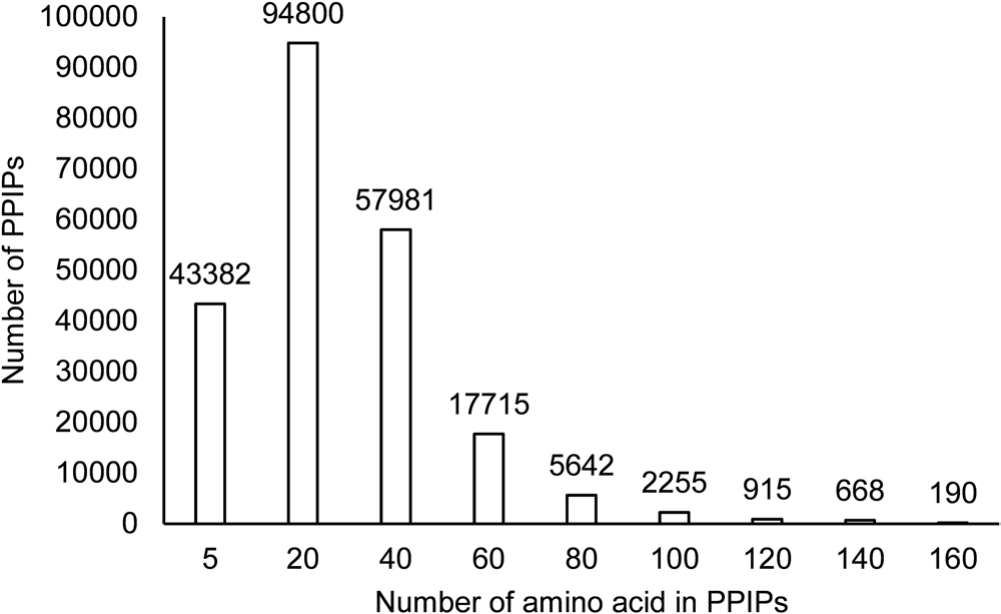
Involvement of amino acids in PPIPs.

The PPIPs in PPInS are very diverse in terms of number of interacting residues they contained. The number of interacting residues in PPIPs varied from as low as a solitary residue to as high as 300. Overall around 4.66 million residues had contributed in the formation of 223714 PPIPs for 111857 protein-protein complexes. The largest PPIP had 310 interacting residues and it belonged to homodimeric structure of Pyruvate Ferredoxin oxidoreductase from *Desulfovibrio africanus*. This was not surprising as homodimer generally tend to have larger interacting interface due to symmetrical structural arrangement around interaction zone. The analysis of PPInS revealed that around 19.39% PPIPs were very short ones and comprised of 1–5 amino acids, 42.37% PPIPs consisted of 6–20 interacting residues, 25.91% PPIPs were reported with 21–40 interacting residues, while only 7.91% PPIPs were made up of 41–60 interacting residues (Fig. 5). Hence, more than 95% of total PPIPs comprised of less than 60 interacting residues and less than 1% PPIPs had more than 100 interacting residues. The PPIPs with interacting 1–5 residues can probably also result from the crystallization conditions, therefore, there is clear literature support that such complexes should be treated with care.

### Solvent accessible surface area (SASA) of PPIPs

**Figure 6:**
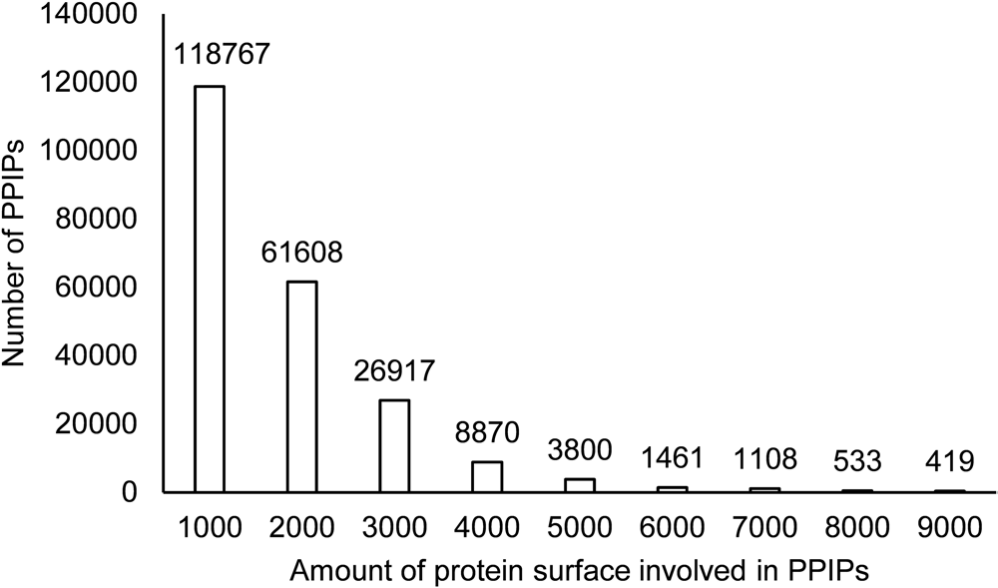
Protein surface area contributed in PPIPs.

The extent of SASA of apo-protein structures shielded from solvent due to the complex formation is an important indicator of the inter-surface interaction affinity. In PPInS a very wide range of SASA precluded from making contacts with the solvent was observed. The SASA of interacting residues in PPIPs varied from very low to very high (approx. 17000 Å^2^). It was observed that 53.08% of PPIPs had less than 1000 Å^2^ SASA, 27.53% PPIPs had 1001–2000 Å^2^, while 12.03% PPIPs were reported with 2001–3000 Å^2^ SASA (Fig. 6). Only 5% of PPIPs had SASA in the range of 4000–17000 Å^2^. The largest PPIP had a SASA of 17409 Å^2^ and it belonged to homodimeric structure of Pyruvate Ferredoxin oxidoreductase from *Desulfovibrio africanus*.

### Hydrophobic nature of PPIPs

**Figure 7:**
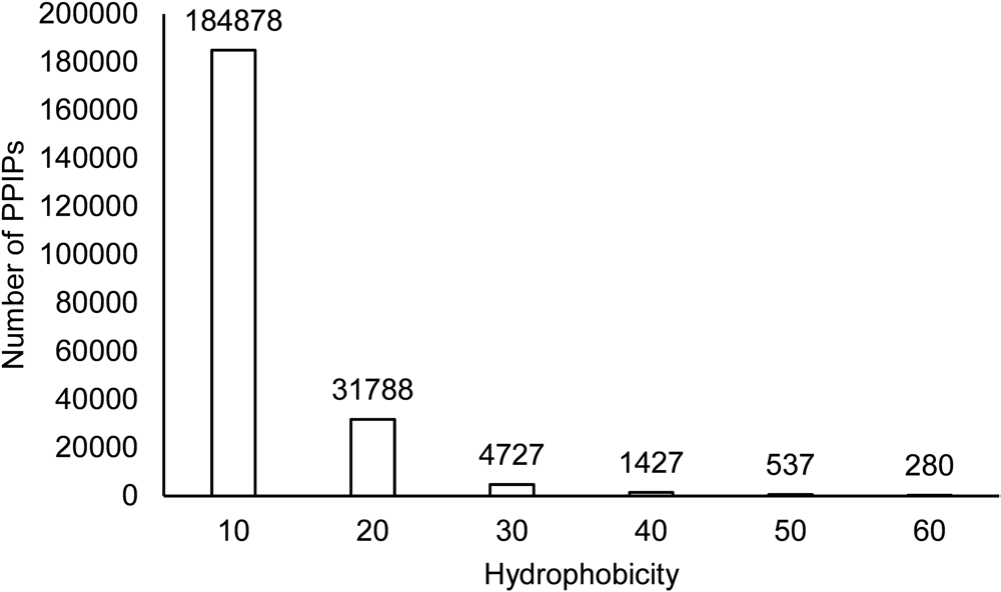
Hydrophobic nature of PPIPs.

The binding energy of protein-protein interaction have a significant contribution of the hydrophobic effect. We determined the diversity in terms of hydrophobicity score of PPIIs covered in PPInS. The hydrophobic index given by Hessa *et al*.^41^ in which amino acid residues are scored on a linear scale from −0.6 (for isoleucine) to 3.49 (for aspartic acid) was used for this purpose. A PPIP with the less hydrophobic score on this scale implies its high hydrophobicity. Observations made from this analysis underlined the hydrophobic nature of PPI sites as it was observed that 82.64% PPIPs were reported with hydrophobicity score of less than 10 and 14.2% PPIPs were reported with hydrophobicity score in the range of 11–20 (Fig. 7). Only 3.16% PPIPs had hydrophobicity score ranging from 21 to the maximum value reported (i.e. 114.41). The most hydrophobic PPIPs were reported in PPII between the protein chains A and B of complex PDB 2B97. The hydrophobicity score reported for these PPIPs was −1.38 and −1.24 for protein chain A and B, respectively.

### Homo and heterodimeric nature of PPIPs in PPIIs

**Figure 8:**
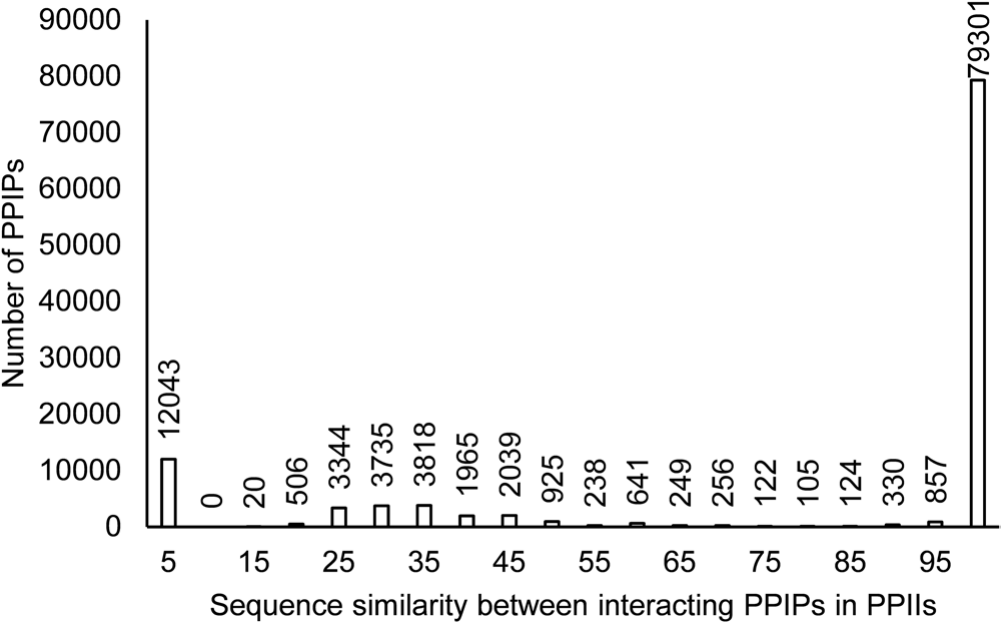
Homo and heterodimeric nature of interacting PPIPs in PPIIs.

The sequence similarity between the interacting PPIPs of PPInS had revealed that 73.26% of total PPIPs were homologous to each other with the sequence similarity score ranging from 75% to the absolute similarity. This is in line with the earlier reports in the literature which state that homodimers are more prevalent in nature. Only 1.34% of PPIPs were reported with 50–75% sequence similarity score, the percentage of the dataset with sequence similarity in the range of 25–50 was 11.15% and the corresponding figure for the sequence similarity between 0–25 was 14.22% (Fig. 8). This is particularly interesting because this could demonstrate that evolutionary mechanism that drives the surface interaction/molecular recognition. A mutation in case of homodimeric proteins is more likely to destabilize the complex rather than strengthening it.

**Figure 9:**
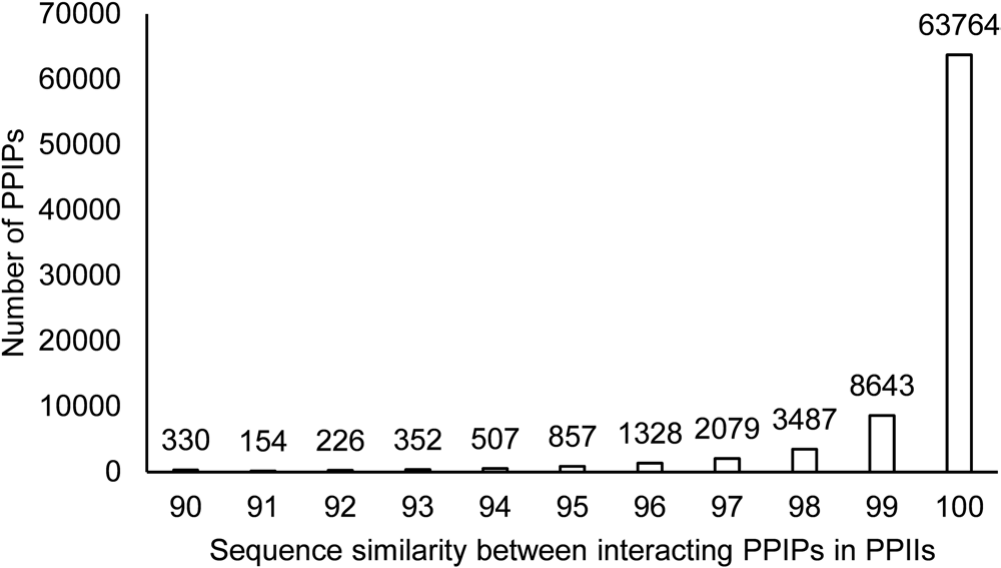
Zoomed-in view of complex destabilization on mutation.

It is also likely that initial events of mutations would only weaken and probably not abolish the homodimeric protein-protein complex and only when the mutations either accumulate beyond a certain threshold (thus changing the very basis of interaction) or when a single mutation is catastrophic in itself for the structural integrity of the complex does the protein-protein interaction partners stop interacting. This is analogous to the trend observed in Fig. 8 depicting that there were around 57% PPIIs with 100% sequence similarity but as the sequence similarity decreased (due to mutations) even by a small fraction, the proteins’ ability to bind with suitable partners decreased drastically to 7% PPIIs with 99% homologous sequences and kept on decreasing thereon (Fig. 9). This demonstrates that a small perturbation in the sequence (thereby the structure) reducing the sequence similarity around 80% minimizes the interaction potential. On the other hand, when the sequence similarity further reduces (say around 75%) the heterodimeric complex formation is favoured.

## Discussion

Proteins are the most important molecular machines whose functional involves a network of dynamic interaction with different molecular surfaces. Understanding these interaction networks help in getting a clearer picture of the cellular processes. A number of experimental and theoretical approaches have been implemented by various research groups for the study of the PPIs. These have resulted in the generation of the very wide amount of data. In this paper, a novel approach to organizing the interaction data is presented in the form of PPInS. Such databases are very useful for the researchers for mining the available information in a logical, systematic and comprehensive fashion. PPInS looks at the protein-protein interaction at the atomic level interaction of the protein surfaces and categorizes the interactions based on the SCOP superfamily (a measure of evolutionary conservation of structure). PPInS has been designed to solely from the experimentally determined structures. To keep the confidence level in the complex structures and to minimize the experimental errors we have restricted the PPInS to include only those structures that have resolution better than 2.5 Å. The interacting partners of protein-protein complexes were demarcated by incorporating the concept of interatomic distances and van der Waals radii of atoms from the interacting proteins of the known protein-protein complex. The PPIIs demarcated in this work has been used in examining the PPIPs with respect to hydrophobicity, solvent accessible surface area, and the number of amino acids contributing in the interacting patch. The homo and heterodimeric nature of the PPIPs in PPIIs was also determined. From which it was observed that 42.37% of total PPIPs were made up of 6–20 interacting residues, 53.08% PPIPs had contributed ≤1000 Å^2^ their protein surface in PPII formation, 82.64% PPIPs were reported with hydrophobicity score ≤10, and 73.26% PPIPs were homologous to each other with the sequence similarity score ranging from 75–100%.

PPInS offers the maximum output in terms of the sheer size and the vast coverage of protein-protein complexes categorized on the evolutionary structural conservation of the interacting proteins. The additional information provided in the context of interacting proteins like graphical representation of the interacting, non-interacting and missing amino acids, SCOP superfamily of interacting proteins, total number of amino acids in interacting proteins, functionality to search for similar protein sequences in PPInS, and three-dimensional structural representation of the protein-protein complex under observation, makes the PPInS more informative.

An unbiased and “proportionally representative” comprehensive dataset is critical for the development of novel PPI prediction tools. In the recent past, vast gamut of methods have been developed for predicting the interacting interface residues, the affinity of protein-protein interaction and conformational changes associated with the binding process. These methods use sequence, protein structure information or more fundamental descriptors for physicochemical characteristics. Such information has been used to generate knowledge-defined prediction methods (based on semantics and syntactic of protein sequence or structure) or for training the machine learning methods based on protein sequences recently proposed by You *et al*.^42^ and Taherzadah *et al*.^43^, structure based approaches of Moal *et al*.^44^ and Xue *et al*.^45^, energy based approaches of Lise *et al*.^46^, evolutionary conservation based methods of Kotlyar *et al*.^47^, Li *et al*.^48^, Huang *et al*.^49^, position-specific scoring matrix based approaches for identification of self-interacting proteins by An *et al*.^50^, and machine learning based approach of Guo *et al*.^51^. The non-redundant database of PPIIs (NRDB) is created to facilitate the developments of such prediction tools. The NRDB is peculiar in having very stringent redundancy control which makes it suited for assessing the various PPI sites parameters (as mentioned above) on a larger scale to draw patterns about the PPI sites and interacting partners.

## Data Availability

PPInS can be accessed from www.cup.edu.in:99/ppins/home.php.

## References

[1] Lo Y, Chen Y, Yang J (2010). 3D-interologs: an evolution database of physical protein-protein interactions across multiple genomes. BMC Genomics..

[2] Veres DV (2015). ComPPI: a cellular compartment-specific database for protein-protein interaction network analysis. Nucleic Acids Res..

[3] Kumar MDS, Gromiha MM (2006). PINT: Protein-protein Interactions Thermodynamic Database. Nucleic Acids Res..

[4] Breitkreutz BJ, Stark C, Tyers M (2003). The GRID: The General Repository for Interaction Datasets. Genome Biol..

[5] Xenarios I (2000). DIP: the Database of Interacting Proteins. Nucleic Acids Res..

[6] Hermjakob H (2004). IntAct: an open source molecular interaction database. Nucleic Acids Res..

[7] Bader GD, Betel D, Hogue CWV (2003). BIND: the Biomolecular Interaction Network Database. Nucleic Acids Res..

[8] Chatr-Aryamontri A (2007). MINT: the Molecular INTeraction database. Nucleic Acids Res..

[9] Ogmen U, Keskin O, Aytuna AS, Nussinov R, Gursoy A (2005). PRISM: Protein interactions by structural matching. Nucleic Acids Res..

[10] Szklarczyk D (2015). STRINGv10: protein-protein interaction networks, integrated over the tree of life. Nucleic Acids Res..

[11] Patil A, Nakai K, Nakamura H (2011). HitPredict: a database of quality assessed protein-protein interactions in nine species. Nucleic Acids Res..

[12] Cukuroglu E, Gursoy A, Keskin O (2012). HotRegion: a database of predicted hot spot clusters. Nucleic Acids Res..

[13] Gunther S, Eichborn JV, May P, Preissner R (2009). JAIL: a structure-based interface library for macromolecules. Nucleic Acids Res..

[14] Jefferson ER, Walsh TP, Roberts TJ, Barton GJ (2007). SNAPPI-DB: a database and API of Structures, iNterfaces and Alignments for Protein-Protein Interactions. Nucleic Acids Res..

[15] Jones S, Thornton JM (1997). Prediction of protein-protein interaction sites using patch analysis. J Mol Biol..

[16] Ofran Y, Rost B (2003). Analysing six types of protein–protein interfaces. J Mol Biol..

[17] Murakami Y, Jones S (2006). SHARP2: Protein-protein interaction predictions using patch analysis. Bioinformatics..

[18] Aytuna AS, Gursoy A, Keskin O (2005). Prediction of protein–protein interactions by combining structure and sequence conservation in protein interfaces. Bioinformatics..

[19] Zhou H, Shan Y (2001). Prediction of protein interaction sites from sequence profile and residue neighbor list. Proteins Struct Funct Genet..

[20] Shen J (2007). Predicting protein-protein interactions based only on sequences information. Proc Natl Acad Sci USA.

[21] Huang Y-A, You Z-H, Chen X, Chan K, Luo X (2016). Sequence-based prediction of protein- protein interactions using weighted sparse representation model combined with global encoding. BMC Bioinformatics..

[22] Fariselli P, Pazos F, Valencia A, Casadio R (2002). Prediction of protein-protein interaction sites in heterocomplexes with neural networks. Eur J Biochem..

[23] Chen X-W, Liu M (2005). Prediction of protein-protein interactions using random decision forest framework. Bioinformatics..

[24] An, J-Y. e*t al*. Robust and accurate prediction of protein self-interactions from amino acids sequence. M*ol Biosyst*. 10.1039/C6MB00599C (2016). 10.1039/c6mb00599c 27759121

[25] Li Z-W, You Z-H, Chen X, Gui J, Nie R (2016). Highly accurate prediction of protein-protein interactions via incorporating evolutionary information and physicochemical characteristics. Int J Mol Sci..

[26] Wang, L. e*t al*. Advancing the prediction accuracy of protein-protein interactions by utilizing evolutionary and ensemble classifier. J *Theor Biol*. 10.1016/j.jtbi.2017.01.003 (2017). 10.1016/j.jtbi.2017.01.003 28088356

[27] Ma B, Elkayam T, Wolfson H, Nussinov R (2003). Protein–protein interactions: Structurally conserved residues distinguish between binding sites and exposed protein surfaces. Proc Natl Acad Sci USA.

[28] Baspinar A, Cukuroglu E, Nussinov R, Keskin O, Gursoy A (2014). PRISM: a web server and repository for prediction of protein-protein interactions and modeling their 3D complexes. Nucleic Acids Res..

[29] Wang L (2017). An ensemble approach for large-scale identification of protein-protein interactions using the alignments of multiple sequences. Oncotarget..

[30] Wei, L. e*t al*. Improved prediction of protein-protein interactions using novel negative samples, features, and an ensemble classifier. A*rtif Intell Med*. 10.1016/j.artmed.2017.03.001 (2017). 10.1016/j.artmed.2017.03.001 28320624

[31] Berman HM (2000). The Protein Data Bank. Nucleic Acids Res..

[32] Fox NK, Brenner SE, Chandonia JM (2014). SCOPe: Structural Classification of Proteins-extended, integrating SCOP and ASTRAL data and classification of new structures. Nucleic Acids Res..

[33] Conte LL, Chothia C, Janin J (1999). The atomic structure of protein-protein recognition sites. J Mol Biol Biol..

[34] Sol delA, Meara PO (2005). Small-world network approach to Identify key residues in protein-protein interaction. Proteins Struct Funct Bioinforma..

[35] Kulharia M, Goody RS, Jackson RM (2008). Information theory based scoring function for the structure based prediction of protein ligand binding affinity. J Chem Inf Model..

[36] Aloy P, Russell RB (2002). Interrogating protein interaction networks through structural biology. Proc Natl Acad Sci USA.

[37] Weigt M, White RA, Szurmant H, Hoch JA, Hwa T (2009). Identification of direct residue contacts in protein-protein interaction by message passing. Proc Natl Acad Sci USA.

[38] Murzin AG, Brenner SE, Hubbard T, Chothia C (1995). SCOP: a structural classification of proteins database for the investigation of sequences and structures. J. Mol. Biol..

[39] Altschul SF (1997). Gapped BLAST and PSI-BLAST: A new generation of protein database search programs. Nucleic Acids Res..

[40] Huang Y, Niu B, Gao Y, Fu L, Li W (2010). CD-HIT Suite: a web server for clustering and comparing biological sequences. Bioinformatics..

[41] Hessa T (2005). Recognition of transmembrane helices by the endoplasmic reticulum translocon. Nature.

[42] You Z-H, Chan KCC, Hu P (2015). Predicting protein-protein interactions from primary protein sequences using a novel multi-scale local feature representation scheme and the random forest. PLoS One..

[43] Taherzadeh G, Yang Y, Zhang T, Liew AW-C, Zhou Y (2016). Sequence-based prediction of protein-peptide binding sites using support vector machine. J Comput Chem..

[44] Moal IH, Jimenez-Garcia B, Fernandez-Recio J (2015). CCharPPI web server: computational characterization of protein-protein interactions from structure. Bioinformatics..

[45] Xue LC, Rodrigues JP, Kastritis PL, Bonvin MA, Vangone A (2016). PRODIGY: a web server for predicting the binding affinity of protein-protein complexes. Struct Bioinforma..

[46] Lise S, Archambeau C, Pontil M, Jones DT (2009). Prediction of hot spot residues at protein-protein interfaces by combining machine learning and energy-based methods. BMC Bioinformatics..

[47] Kotlyar, M. e*t al*. In silico prediction of physical protein interactions and characterization of interactome orphans. N*at Methods*. 10.1038/nmeth.3178 (2014). 10.1038/nmeth.3178 25402006

[48] Li Z-W (2017). Accurate prediction of protein-protein interactions by integrating potential evolutionary information embedded in PSSM profile and discriminative vector machine classifier. Oncotarget..

[49] Huang Y-A, You Z-H, Chen X, Yan G-Y (2016). Improved protein-protein interactions prediction via weighted sparse representation model combining continuous wavelet descriptor and PseAA composition. BMC Syst Biol..

[50] An J-Y (2016). Identification of self-interacting proteins by exploring evolutionary information embedded in PSI-BLAST-constructed position specific scoring matrix. Oncotarget..

[51] Guo H, Liu B, Cai D, Lu T (2018). Predicting protein-protein interaction sites using modified support vector machine. Int J Mach Learn Cybern..

